# Foot care knowledge and practices among Japanese nurses and care workers in home care and adult service center: a cross- sectional study

**DOI:** 10.1186/s12912-020-00467-1

**Published:** 2020-08-06

**Authors:** Kashiko Fujii, Takuyuki Komoda, Atsuko Maekawa, Mariko Nishikawa

**Affiliations:** 1 Graduate School of Medicine, School of Health Sciences, Nagoya University, 1-1-20 Daiko-Minami, Higashi-ku, Nagoya City, Aichi Prefecture 461-8673 Japan; 2 Department of Plastic and Reconstructive Surgery, Gifu Heart Center, 4-14-4 Yabuta Minami, Gifu City, Gifu Prefecture 500-8384 Japan; 3 Faculty of Nursing, Shubun University, 6 Nikko-cho, Ichinomiya-City, Aichi Prefecture 491-0938 Japan; 4 Graduate School of Nursing, University of Human Environments, 3-220 Ebata-Cho, Obu City, Aichi Prefecture 4740035 Japan

**Keywords:** Foot care, Knowledge, Practice, Nurse, Care worker

## Abstract

**Background:**

Foot care knowledge and practices among nurses and care workers in the community greatly impact foot health maintenance and prevention of foot-related problems among older people. This study aimed to explore and examine the current foot care knowledge, practices, and perceptions among nurses and care workers at home care and adult day service center, along with their demographic characteristics and daily care for clients.

**Methods:**

This study analyzed 232 randomly selected front-line nurses and care workers working at home care or adult day service center in one of the selected cities, Aichi Prefecture, Japan. Data were obtained using questionnaires and subsequently analyzed using descriptive statistics, t-tests, Chi-square tests, Wilcoxon rank-sum tests, and Spearman’s rank correlation tests.

**Results:**

Among the 305 surveyed, 232 (62 nurses; 170 care workers) provided data. Although 57 nurses (91.9%) and 142 care workers (83.5%) showed interest in foot care, 33 nurses (53.2%) and 133 care workers (78.2%) stated that foot care education was insufficient. Knowledge and practice scores were associated with working status.

Higher accuracy differences in the early detection of foot problems and skin tears on lower limbs in knowledge category were observed between nurses and care workers. The nurses as well as the care workers had low accuracy rates of knowledge questions regarding the use of shoes and socks subscale.

For practice, both nurses and care workers had low mean scores for checking client’s shoes (2.0/5.0 and 2.1/5.0, respectively), method for reducing ingrown nail pain (2.6/5.0 and 1.9/5.0, respectively), and opportunity for discussing foot care with others (2.7/5.0 and 2.2/5.0, respectively). A significant correlation between knowledge and practice scores was observed among nurses (0.331, *p* < 0.05) and care workers (0.339, *p* < 0.001).

**Conclusions:**

Despite the presence of several barriers toward enhanced care delivery to clients needing it most, nurses and care workers clearly understood the importance of foot care. These findings indicate that foot care should be focused by nurses and care workers to improve the knowledge and practice of foot care and to suggest future implications that efficient and understandable tools are needed considering their current working situation.

## Background

As the population worldwide continues to rapidly age [1], growing demands and expanding costs for health care, particularly for geriatric care, have been greatly concerning for many countries. To reduce elderly care expenditure and hospital loading in Japan, the Japanese government has promoted the use of home health care services [2]. Indeed, studies have shown an increasing trend in the number of older people receiving home care services [3], with nurses and care workers playing a key role in maintaining the health of older individuals at home or within a community.

Foot problems have been one of the most prevalent concerns among older individuals. As the body ages, structural, functional, and physiological changes within the circulatory, skeletal, nervous, and dermatological systems can cause a range of foot problems, including toe nail problems, toe deformities, corns, calluses, fungal infections, cracks, fissures, macerations, and edema [4]. These conditions can lead to foot pain [5], which has been associated with reduced mobility and balance and increased risk for gait disorders, falls, and depression [6–10]. Many older people are frail and live alone with limited access to medical care. Moreover, reduced vision, physical function, and manual dexterity [11], muscle alternations, [12] are some of the factors that inhibit their ability for foot self-care. Abdullah and Abbas [13] stated that nail problems are common among older adults and are often overlooked by primary caregivers despite their various physical and physiological characteristics.

The increasing number of older people within communities throughout Japan provides greater opportunities for nursing care at home, at day care service centers, or at day care centers offering rehabilitation [14]. Japan has two insurance programs that allow older people access to medical or nursing care (Appendix 1) according to their condition (i.e., some are vulnerable and bedridden, while others have good health). After matching procedural demand and supply, older people subsequently receive the necessary medical or physical care, as well as assistance with activities of daily living, from nurses and care workers providing home care services. Both nurses and care workers have equally high opportunities for physically contact with older individuals. Moreover, care workers’ subjective observations regarding the client’s physical and emotional condition are often shared with nurses and care managers in Japan. Previous studies in other countries have investigated nursing assistants’ detection of early signs of infection or acute or chronic illness in nursing homes [15–18]. Thus, both nurses and care workers function autonomously in detecting foot problems, making decisions regarding foot care, and reporting to other health care professionals.

Foot problems have been widely studied internationally. However, literature regarding foot conditions and foot care has predominantly focused on diabetes [19], while limited studies have been available on foot care knowledge, practices, and perceptions among nurses and care workers in home or community settings. Considering that podiatry is not considered as a specialty in Japan, foot care knowledge and skills can be obtained at private schools that charge relatively high tuition fees. Moreover, the lack of foot care education within academic curriculum may lead to insufficient foot care knowledge and practices among nurses and care workers. The present study aimed to explore and examine the current knowledge, practices, and perceptions among nurses and care workers in in-home service providers, along with their demographic characteristics and daily number of clients. This survey can serve as a reference for the future development of more effective foot care tools for nurses and care workers in home or community settings. The present study hypothesizes the following:

1. Both nurses and care workers show interest in learning foot care but may perceive to have insufficient education on and time for foot care and display a lack of confidence in the same.
2. No significant differences in foot care knowledge and practices exist between nurses and care workers regardless of working status or experiences due to the fundamental lack of foot care education in Japan.
3. Nurses obtain and demonstrate better foot care knowledge and practices related to vascular, neurologic, and skin disorders compared to care workers due to differences in educational curriculum.

## Methods

### Research design

This was a cross-sectional study using random cluster sampling. Target participants comprised nurses and care workers from 35 different in-home service providers [14] in one of the selected cities, Aichi Prefecture, Japan. Data were collected from July to August 2019.

### Instruments

Data were collected via questionnaires called *Kashiko XJP*, which were developed specifically for nurses and care workers included in this study. The questionnaires consisted of the following: (1) questions on demographic characteristics, daily number of clients or number of clients with foot problems, and perceptions regarding foot care; (2) 30 questions on foot care knowledge; and (3) 20 questions on foot care practices.

### Sampling and participants

As of 2018 (2017 survey), there are 147,827 nurses and 747,370 care workers working at home care and adult day service centers in Japan [20] (main author’s calculation based on the dataset). This study targeted nurses and care workers working at centers providing home care, home nursing, adult day care centers including day care services, or day care services with rehabilitation. Registered nurses (RN), licensed practical nurses (LPN), certified care workers, and noncertified care workers with different types of qualifications were included. In this paper, the term “LPN” is used that is “assistant nurse” in accordance to the Japanese law translation [21] and the job profile might slightly differ from that of “LPN” defined by the United States of America.

In Japan, the qualification and experience of nurses were as follows: RN and LPN. Both RN and LPN are medical professions that provide medical treatment and assist in examination. The major differences in RN and LPN are in terms of the place that issues the qualification, and the qualification requirements. The license for a registered nurse is given by the Minster of Health, Labour and Welfare, whereas that for a LPN is given by the Prefectural Governor. According to the Act, LPNs perform their job under the direction of a physician, dentist, or nurse [21].

Care workers were not allowed to provide medical conduct. They provided nursing services such as assisting in oral care, bathing, meal, going to the toilet, e.g., to sustain the daily needs of clients at facilities. The care workers include certified care workers who are qualified after having passed the certified care workers examination and noncertified care workers. Many noncertified care workers have a certificate of novice training or practitioner training. The major differences between certified and noncertified care workers are the contents and duration of training they have undergone to achieve the certification. Their tasks are similar; however, certified care workers have more tasks of consultation or providing instruction to clients and their family.

Other countries identify care workers as “nursing assistants” nursing aides [15, 18]. The sample size was calculated based on a 95% confidence interval and 5% margin of error using a sample size chart. Accordingly, an initial sample size of 530 nurses and care workers was targeted with a nurse/care worker ratio of 20%/80% (1:4) and a 10% participant rejection rate. The inclusion criteria were nurses and care workers who (1) worked part time or full time, (2) provided physical care, including but not limited to walking assistance, diaper changing, bathing assistance, exercise promotion, and oral care assistance, and (3) worked for centers that never participated in any other foot care programs besides the current study. The Ministry of Health, Labour and Welfare (MHLW) provided a list of in-home service providers in one of the selected cities, Aichi Prefecture, Japan, among which 350 centers were initially randomly selected via computer for our study. An invitation mail and a postcard with a check box indicating the level of willingness to participate in the survey and the number of possible participants in each center were sent to all randomly selected centers. However, the target number of replies was not reached. Thus, 100 day care service centers were added using the same methodology. Overall, postcards from 78 centers were returned, among which 46 were willing to participate in the study and indicated the possible number of study participants. After confirming participation via telephone and personal visit by the main author (KF), questionnaires were sent to the 46 centers (305 nurses and care workers). Among the 305 nurses and care workers who had received questionnaires, 232 (76%) from 35 centers responded with written approval (62 nurses and 170 care workers) (Table 1).

**Table 1:** Number and type of providers contacted and replies received

Provider type	Number of centers contacted	Questionnaires sent	Questionnaires returned	Collection rate
Day care service centers	370	36	25	69%
Day care centers offering rehabilitation	30	2	2	100%
Home nursing centers	20	6	6	100%
Home care centers	30	2	2	100%
Total number of centers	450	46	35	76%

### Development of foot care knowledge and practice questionnaires

The questionnaires used herein were developed in Japan and were initially based on foot care knowledge and practice questionnaires for nurses in Finland [11, 19]. However, given the inclusion of care workers in the present study and the geographical differences in the standards of care between Japan and Finland, modifications to these questionnaires were required in order to address the purpose of this study. Therefore, a new questionnaire was developed utilizing three phases.

#### Phase one: draft creation

The item pool (Table 2) and subscales for the draft questionnaires were created based on a thorough literature review of 339 studies. Personal face-to-face contact or e-mail correspondence with foot researchers, including a foot care specialist (IY), and the main author’s (KF) clinical experiences in nursing and foot care also contributed to the creation of the draft. Draft questionnaires consisted of 51 questions on knowledge covering seven subscales (Nail, Skin, Vascular and Neurologic Disorder, Toe and Arch, Infection, Shoes and Socks, and Sedentary Behavior) with three possible answers (yes, 1 point; no, 0 points; and I do not know, 0 points) and 45 questions on practices covering six subscales (Skin Assessment, Nail, Skin, Hygiene, Movement and Toe Exercise, and Consultation) with five possible answers (strongly relevant, 5 points; more relevant, 4 points; neutral, 3 points; less relevant, 2 points; and not relevant, 1 point). Other specific questions included demographic characteristics [age, sex, profession, part-time or full-time employment, working experience in the current profession, working experience in the current center (this question was later removed)], the daily number of clients, number of clients with foot problems in the previous month, and perceptions regarding foot care (interest in foot care, impression on the current foot care education, confidence in foot care, source of foot care knowledge, opinion on foot care manuals, sufficient time for foot care, willingness to learn more about foot care, and self-use of toe socks).

**Table 2:** Item pool for draft questionnaires

Foot care guidelines by the Ministry of Health, Labour and Welfare	
Checking physical conditions (e.g., blood pressure, pulse) before starting foot measurements and exercises	
Morphological, functional, and physiological structures and roles of the toe, foot, and leg; demographic and social changes in Japan	
Fall risks at home	
Type of foot problem	
Toenail problems	
Foot skin problems	
Foot vascular problems and assessment	
Foot neurologic problems and assessment	
Diseases causing foot problems	
Difference and finding Differentiating between corns and calluses	
Foot skin and toenail fungus	
Characteristics of body change and anatomical changes among elderly individuals	
Foot skin conditions among elderly individuals	
Influence of sedentary behavior on the body	
Foot muscles and capillaries	
Influence of shoes and socks on toe and foot problems	
Selection of shoes and socks	
Types of foot massage	
Sitting and standing posture	
Foot hygiene	
Procedures for nail cutting with nail filing	
Reducing corns and calluses through foot filing	
Appropriate procedure for nail cutting	
Cotton packing to reduce ingrown nail pain	
Taping to reduce ingrown nail pain	
Locating the posterior tibial artery of the foot	
Identifying neurologic impediments	
Infection control	
Toe and foot exercises	
Ointment application	
Consultation and referral to doctors or other health professionals	

#### Phase 2: content validity

Four experts, including a nurse researcher, a previously contacted foot care specialist (IY), and two researchers specializing in toe movement, as well as two nurses and a care worker with field experience, were contacted to review the draft questionnaires and provide their opinion regarding the usability and necessary adjustments at this stage. The validity of draft questionnaires was evaluated using two processes. Firstly, questionnaires were mailed to eight experts and hand-delivered to one expert for evaluation using the Content Validity Index (CVI), an internationally recognized scale and the most widely used approach for assessing content validity [22]. These experts included a foot care specialist (*n* = 1), nurses with extensive foot care experience (*n* = 3), a nursing academic researcher (*n* = 1), physical therapists specializing in foot and toe therapy (*n* = 2), a surgeon with extensive knowledge on foot physiology (*n* = 1), and a doctor with extensive knowledge on wound care (*n* = 1). Secondly, a panel of experts consisting of a surgeon, three nurse researchers with expertise in foot research, a foot care specialist, and a nurse with foot care expertise, discussed the clarity, wording, relevance, and necessity of each question. During panel discussions, the mean CVI scores provided by the nine experts who initially evaluated the questionnaires were used as reference. After further refinement, the questionnaires included 33 questions on foot care knowledge and 25 on foot care practices, along with 16 other specific questions regarding demographic characteristics, daily number of clients, and perceptions regarding foot care.

#### Phase 3: pilot study

A pilot study was conducted among 100 nurses and care workers from in-home service providers and geriatric facilities excluding hospitals, among whom 87 (73%) responded to knowledge and practice questions, respectively. Their answers were subsequently analyzed using SPSS 24 (SPSS Inc., Chicago, IL) to determine their validity and reliability. Descriptive statistics was used to describe data characteristics and questionnaire scores. Questions regarding knowledge were then thoroughly reviewed based on accuracy rates. Five questions on practices demonstrated the ceiling effect and were thus removed. Cronbach’s alpha values, which were calculated to evaluate internal consistency, were between 0.5 and 0.7, with 0.70 being considered an acceptable value. However, acceptable Cronbach’s alpha values vary between researchers [23]. Thereafter, the final format of the self-administrated questionnaires, which consisted of 50 questions on knowledge (30 questions across seven subscales) and practices (20 questions across six subscales) along with 15 questions on demographic characteristics, daily number of clients, and perceptions regarding foot care, was established.

### Data analysis

Data were recorded by two separate teams working simultaneously with the same information without being observed by the researchers using an outsourcing company. Data were then categorized into two groups, nurses and care workers, and subsequently analyzed using SPSS 24. Descriptive statistics, χ^2^ tests, and the Wilcoxon rank-sum test were utilized for data analysis. Content validity was analyzed using the ceiling effect with means ± standard deviations (SDs), while reliability was analyzed by calculating Cronbach’s alpha values. Spearman’s rank correlation was used to determine the correlation (1) between knowledge and practice scores and gender, working experience in the current profession, and number of clients cared for per day and (2) between knowledge scores and practice scores. The association between age and working status and knowledge and practice scores was analyzed using Student’s t-test.

## Results

### Demographic characteristics, daily number of clients, and perceptions on foot care

**Fig. 1:**
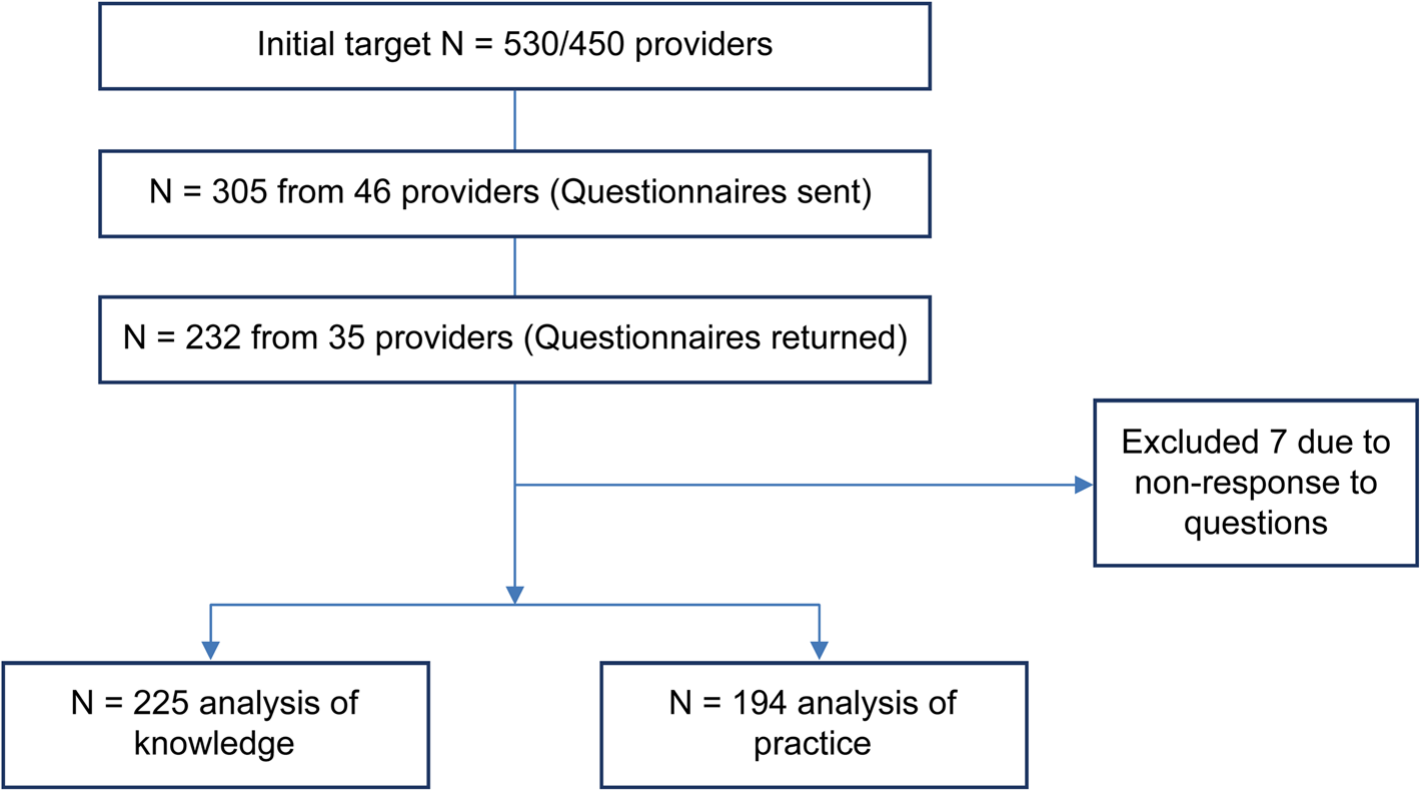
Trial profile

Among the 232 respondents, (52 RNs, 10 LPNs, 98 certified care workers, and 72 noncertified care workers with different types of qualifications), 225 were ultimately analyzed after excluding two nurses and five care workers for non-response to questions (Fig. 1).

Effect size was calculated using EZR for both knowledge and practice: Formula = (mean scores for nurses − mean scores for care workers)/[(SD for nurses + SD for care workers)/2]. The number of nurses and care workers used in the formula included those who answered all questions. Although an effect size of 0.6 was calculated, a statistical power of 0.95 was obtained. Therefore, the final number of participants (225) was acceptable despite the initial target being 530.

The ratio between nurses and care workers was 1:3, which was quite close to the initially targeted ratio of 1:4. Complete results for demographic characteristics, daily number of clients, and perceptions regarding foot care are presented in Tables 3 and 4.

**Table 3:** Demographic characteristics, daily number of clients, and perceptions on foot care

*N* = 232				
Items	Category	Nurses^**1**^ (***n*** = 62)	Care workers^**2**^ (***n*** = 170)	***p*** value
		***n*** (%)	***n*** (%)	
Sex	Male	2 (1.6)	29 (17.1)	0.002**
	Female	60 (98.4)	141 (82.9)	
Profession	Registered nurses	52 (83.9)	N/A	0.000***
	Licensed practical nurses	10 (16.1)	N/A	
	Certified care workers	N/A	98 (57.6)	
	Noncertified care workers	N/A	72 (42.4)	
Work status	Part time	34 (54.8)	64 (37.3)	0.017*
	Full time	28 (45.2)	106 (62.7)	
Number of clients cared for per day	1–5	29 (46.8)	14 (8.4)	0.000***
	6–10	7 (11.3)	37 (22.2)	
	11–20	13 (21.0)	53 (31.7)	
	21–30	10 (16.1)	34 (20.4)	
	31+	3 (4.8)	29 (17.4)	
Interest in foot care	Yes	57 (91.9)	142 (83.5)	0.105
	No	5 (8.1)	28 (16.5)	
Impression on current foot care education	Sufficient	2 (3.2)	1 (0.6)	0.000***
	Neutral	27 (43.5)	36 (21.2)	
	Insufficient	33 (53.2)	133 (78.2)	
Confidence in foot care	Confident	3 (4.8)	1 (0.6)	0.000***
	Neutral	31 (50.0)	43 (25.4)	
	Not confident	28 (45.2)	125 (74.0)	
Source of foot care knowledge	Not obtained	10 (17.2)	72 (43.4)	0.000***
	Work	18 (31.0)	24 (14.5)	0.005**
	Outside work	15 (25.9)	22 (13.3)	0.026*
	Journal/magazine	19 (32.8)	26 (15.7)	0.005**
	Internet	16 (27.6)	23 (13.9)	0.018*
	Colleagues	18 (31.0)	59 (35.5)	0.534
	Television	13 (22.4)	22 (13.3)	0.098
Opinion on care manuals	Required	48 (78.7)	128 (75.7)	0.472
	Neutral	13 (21.3)	37 (21.9)	
	Not required	0 (0.0)	4 (2.4)	
Sufficient time for foot care	Agree	19 (31.7)	26 (15.6)	0.007**
	Disagree	41 (68.3)	141 (84.4)	
Willingness to learn more about foot care	Yes	53 (85.5)	120 (71.0)	0.057
	Neutral	9 (14.5)	44 (26.0)	
	No	0 (0.0)	5 (3.0)	
Self-use of toe socks	Used	12 (19.4)	49 (29.0)	0.274
	Neutral	9 (14.5)	17 (10.1)	
	Not used	41 (66.1)	103 (60.9)	

^1^Chi-square distribution

*p < 0.05

**p < 0.01

***p < 0.001

**Table 4:** Age, working experience, and number of clients with foot problems cared for

*N* = 232							
Item	Nurses			Care workers			***p*** value
	n	Mean	SD	n	Mean	SD	
Age	62	51.2	12.2	170	47.8	11.6	0.062
Working experience in the current profession	58	23.2	12.2	162	9.2	5.7	0.001***
Number of clients with foot problems cared for within past month.	57	7.9	8.3	165	9.5	11.6	0.346

*SD* standard deviation

Student’s t-test *p < 0.05

**p < 0.01

***p < 0.001

Nurses and care workers had a mean age of 51.2 (SD 12.2) and 47.8 (SD 11.6) years, respectively. Nurses and care workers provided care to a mean of 7.9 (SD 8.3) and 9.5 (SD 11.6) clients with foot problems in the last month, respectively (Table 4). Moreover, 34 (54.8%) and 28 (45.2%) nurses worked part time and full time, while 64 (37.3%) and 106 (62.7%) care workers worked part time and full time, respectively (Table 3).

About 29 (46.8%) nurses cared for 1–5 clients a day, whereas 37 (22.2%) care workers cared for 6–10 clients and 53 (31.7%) care workers cared for 11–20 clients per day. An overwhelming majority of nurses (57, 91.9%) and care workers (142, 83.5%) were interested in foot care. A total of 33 (53.2%) nurses and 133 (78.2%) care workers thought foot care education was lacking. Only 3 (4.8%) nurses and 1 (0.6%) care worker had confidence in their foot care practices. More than three quarters of nurses (48, 78.7%) and care workers (128, 75.7%) thought foot care guidelines were necessary. Furthermore, 41 (68.3%) nurses and 141 (84.4%) care workers thought they did not have sufficient time for foot care, whereas 53 (85.5%) nurses and 120 (71.1%) care workers were interested in learning more about foot care. A total of 59 care workers (35.5%) obtained foot care knowledge from colleagues, whereas 83% of nurses obtained it from various sources. Meanwhile, 72 care workers (43.4%) did not obtain foot care knowledge from any sources. A total of 12 (19.4%) nurses and 49 (29.0%) care workers used toe socks.

As shown in Table 5, practice scores were significantly associated with work status (part time and full time) for both nurses and care workers. Mean practice scores were higher among full-time providers than part-time providers in both groups.

**Table 5:** Correlation between knowledge and practice scores and demographic characteristics and daily number of clients

	**Total knowledge score**				**Total practice score**			
	**n**	**Spearman’s correlation coefficient**		***p*value**	**n**	**Spearman’s correlation coefficient**		***p*value**
Nurse (*n* = 62)								
Age	57	0.087		0.521	50	0.109		0.451
Working experience in the current profession	56	0.012		0.929	48	0.013		0.928
Number of clients cared for per day	60	−0.073		0.580	52	−.301		0.030*
Care workers (*n* = 170)								
Age	164	−0.080		0.306	142	0.117		0.165
Working experience in the current profession	158	0.069		0.387	137	0.332		0.000***
	Total knowledge score				Total practice score			
	n	Mean	SD	*p* value	n	Mean	SD	*p* value
Nurse (*n* = 62)								
Male	0	–	–	–	0	–	–	–
Female	59	25.1	2.4		51	65.5	13.6	
Work status: part time	33	25.3	2.5	0.708	25	61.4	13.8	0.023*
Work status: full time	27	25.0	2.4		27	69.9	12.3	
Care workers (*n* = 170)								
Male	26	22.6	5.7	0.875	25	55.1	13.4	0.26
Female	139	22.4	4.6		117	58.3	13.1	
Work status: part time	63	21.8	5.2	0.195	51	53.1	12.4	0.001**
Work status: full time	101	22.8	4.4		90	60.5	12.9	

Age, working experience in the current profession, and number of clients for per day were analyzed using “test for no correlation”

Sex and working status were analyzed using Student’s t-test

*p < 0.05

**p < 0.01

*** *p* < 0.001

Mean practice scores were found to be associated with the number of clients cared for per day among nurses and working experience in the current profession among care workers.

### Foot care knowledge

Among the 232 participants included, 225 (96%) completely answered all questions regarding knowledge, while 7 (4%) did not answer parts of the questions. Table 6 details the accuracy rates of the answers. Significant differences were observed in the early detection of foot problems (Vascular and Neurologic 2) and skin tear on lower limbs (Skin 5) between nurses and care workers, with an accuracy difference of 34.3 and 25.5%, respectively, which was highest among knowledge items. Accuracy rates in both groups were low for the Shoes and Socks subscale.

**Table 6:** Accuracy rates for knowledge questions according to profession

*N* = 225							
Subscales	Item	Nurses		Care workers		Differences	***p*** value
		N	% Accuracy	n	% Accuracy		
Nail 1	Cutting a toenail shorter than the tip of the toe may cause a curly nail, and/or an ingrown nail.	58	96.7	125	75.8	20.9	0.000**
Nail 2	A toenail can be cut easier after soaking nails in warm water for 5–10 min.	57	95.0	148	89.7	5.3	0.216
Nail 3	Toenails protect the end of the foot and support body weight when walking.	55	91.7	120	72.7	19	0.003**
Nail 4	When a nail is yellowed and rough, a fungal infection is the suspected cause.	55	91.7	146	88.5	3.2	0.494
Nail 5	The color of the nail can be used as barometer of general health.	57	95.0	154	93.3	1.7	0.647
Skin 1	Moisturizer should be applied immediately after taking a bath.	50	83.3	129	78.2	5.1	0.397
Skin 2	Corns and calluses have the same meaning.	45	75.0	103	62.4	12.6	0.079
Skin 3	Repeated friction and stimulation cause the keratin in the sole of the foot to become thicker.	49	81.7	118	71.5	10.2	0.124
Skin 4	Skin tear on an older person’s upper arms or elbow joints are often produced when a caregiver adds extra pressure when assisting movement.	38	63.3	66	40.0	23.3	0.002**
Skin 5	Skin tear on lower limbs often occurs by coming into contact with appliances such as footrests.	52	86.7	101	61.2	25.5	0.000***
Skin 6	Because there are no sebaceous glands on the soles, oil is unavailable, and the sole becomes dry easily.	38	63.3	95	57.6	5.7	0.437
Vascular and Neurologic 1	If the client suffers from severe diabetes, foot sensitivity is reduced and pain may not be noticed even though he/she was injured.	60	100.0	143	86.7	13.3	0.003**
Vascular and Neurologic 2	When only one foot only suddenly becomes cold, blood vessels may be blocked by blood clots.	58	96.7	103	62.4	34.3	0.000***
Vascular and Neurologic 3	Small wounds on an older person may develop into an ulcer if left without treatment.	58	96.7	134	81.2	15.5	0.004**
Vascular and Neurologic 4	Signs of infection are flares (reddish tinge), swelling, pain and a feeling of heat.	56	93.3	134	81.2	12.1	0.027*
Vascular and Neurologic5	Even though pain is felt on one foot after a period of walking, it will go away after rest. Consequently, there is no need to worry.	52	86.7	144	87.3	−0.6	0.905
Toe and Arch 1	There is no relationship between foot or toenail deformation, and pain in the waist or neck.	51	85.0	121	73.8	11.2	0.078
Toe and Arch 2	When one of the three arches on the foot collapses, various problems occur.	52	86.7	112	67.9	18.8	0.005**
Toe and Arch 3	A stiff ankle is more likely to make a person stumble or fall.	59	98.3	137	83.0	15.3	0.002**
Toe and Arch 4	Toe deformity influences the muscular strength of lower limbs.	58	96.7	152	92.1	4.6	0.227
Toe and Arch 5	Toe flexor exercise increases the calf muscle pump function of lower limbs.	60	100.0	128	78.0	22.0	0.000***
Infection 1	Fungal bacteria can be removed from the nail clippers using alcohol.	43	71.7	126	76.4	−4.7	0.471
Infection 2	When medical appliances are shared among clients without sufficient sterilization, infection spreads.	59	98.3	155	93.9	4.4	0.176
Infection 3	The bucket used for foot baths is cleaned only by rinsing with hot water.	45	75.0	126	76.4	−1.4	0.832
Shoes and Socks 1	The client’s shoes have approximately 1–1.5cms space, measured from the longest toe, and allow the toes to move freely.	34	56.7	83	50.3	6.4	0.398
Shoes and Socks 2	Corns and calluses are not influenced by the type of socks worn.	31	51.7	97	58.8	−7.1	0.340
Shoes and Socks 3	Shoe sizes are not absolute and vary by a maker.	53	88.3	142	86.1	2.2	0.657
Shoes and Socks 4	Shoes with a well-fixed heel prevent foot slippage.	29	48.3	98	59.4	−11.1	0.139
Sedentary Behavior 1	Walking for 1 h a day is enough to compensate for long sedentary periods.	54	90.0	146	88.5	1.5	0.749
Sedentary Behavior 2	Falls that happen when an older person moves from sitting to standing can be prevented by planning from the care worker.	44	73.3	117	70.9	2.4	0.722

Chi-square test *p < 0.05

**p < 0.01

*** *p* < 0.001

Among the 232 participants, 225 (96%) answered all questions regarding knowledge

Answer: yes = 1, no = 2, I do not know = 3

### Foot care practices

Result for questions regarding practices among nurses and care workers are presented in Table 7. Among the 232 participants, 194 (84.4%) answered all 20 questions regarding practices. The results demonstrated some differences in foot care practices between nurses and care workers. Accordingly, significant differences between nurses and care workers were found in the daily assessment of clients’ feet, assessment of the skin between the toes and on the heel, use of nippers, method for reducing ingrown nail pain, the use of a grinder, drying of the skin between the toes, the use of soap, awareness regarding foot baths, and talking about foot care with other staff members. Both groups had close mean scores for the three items on Movement and Toe Exercises.

**Table 7:** Mean scores for questions regarding practices according to profession

*N* = 225								
Item	Item content	Nurses (62)			Care workers (170)			***p*** value
		n	Mean	SD	n	Mean	SD	
Skin Assessment 1	I (as care giver) check the clients’ feet every day.	59	3.1	1.0	158	2.7	1.1	0.007**
Skin Assessment 2	When I check each foot, the skin between the toes and on the heel is included.	59	3.1	1.1	158	2.5	1.1	0.000***
Skin Assessment 3	I check the clients’ shoes before they wear or take off their shoes.	58	2.0	0.8	156	2.1	0.9	0.631
Nail 1	When I clip the clients’ nails, they are clipped straight with a curve at the corners.	58	3.6	1.2	155	3.3	1.3	0.113
Nail 2	I always use the nipper when I cut the clients’ nails.	59	3.2	1.5	156	2.4	1.5	0.000***
Nail 3	When there is a slight ingrown nail, I know the method to reduce pain by taping and packing with cotton.	58	2.6	1.5	157	1.9	1.3	0.000***
Nail 4	I use a nail file or grinder to reduce the thickness of nails that require this treatment.	59	3.0	1.4	157	2.3	1.4	0.001***
Nail 5	Sterilizing method is the same within an institution after the use of a nipper.	59	2.7	1.4	155	3.1	1.5	0.070
Skin 1	After I wash the clients’ feet, the area between the toes is dried thoroughly.	59	3.7	1.3	156	3.0	1.3	0.000***
Skin 2	When heels are cleaned every day, they become cleaner.	57	3.5	1.2	154	3.5	1.2	0.952
Skin 3	Moisturizer is used on dry feet because dryness reduces the barrier function of skin.	59	4.2	1.0	158	3.9	1.2	0.140
Skin 4	I sometimes apply Vaseline or an ointment to the skin without first wiping away previous excess Vaseline or ointments.	58	2.9	1.4	158	2.5	1.4	0.078
Hygiene 1	It is beneficial to bathe in acidic bubble soap.	58	4.0	1.1	157	3.6	1.2	0.036*
Hygiene 2	Bathing opens the skin’s pores more effectively than showers; therefore, a bath is more effective in removing dirt.	58	4.0	1.2	156	3.9	1.1	0.842
Hygiene 3	I understand the purpose, method and awareness points for care of a foot bath.	59	3.7	0.9	157	2.8	1.1	0.000***
Movement and Toe Exercise 1	I provide advice to clients when they stand from a chair.	59	3.5	1.0	157	3.4	1.1	0.746
Movement and Toe Exercise 2	I always promote toe exercises to clients.	59	3.6	1.2	157	3.4	1.3	0.423
Movement and Toe Exercise 3	I encourage clients to stand when they have been sitting for more than 1 h.	57	3.2	1.2	158	3.0	1.2	0.303
Consultation 1	I have an opportunity to talk about foot care with other staff members.	59	2.7	1.4	158	2.2	1.1	0.005**
Consultation 2	I always consult with others regarding which doctor or specialist the client should visit.	59	3.2	1.3	158	2.2	1.2	0.000***

*SD* standard deviation

Mann–Whitney U *p < 0.05

**p < 0.01

*** *p* < 0.001

Five answers: strongly relevant = 5, more relevant = 4, neutral = 3, less relevant = 2, not relevant = 1 (points)

Among the 225 participants, 194 answered all of the questions

Nurses and care workers had low mean scores for checking clients’ shoes (2.0 and 2.1, respectively), method for reducing ingrown nail pain (2.6 and 1.9, respectively), and talking about foot care with other staff members (2.7 and 2.2, respectively). However, nurses and care workers had relatively high means scores for skin moisturizing (4.2 and 3.9, respectively) and bathing effects (4.0 and 3.6, respectively).

Although a ceiling effect was observed for items Nail 3, Skin 3, and Hygiene 2, they were statistically acceptable given their SD of 1.4, 1.1, and 1.1, respectively (Table 8).

**Table 8:** Observed ceiling effects on mean scores for questions regarding practices (nurses and care workers)

Items	Mean	SD	M – SD	M + SD	Ceiling effect
Skin Assessment 1	2.8	1.1	1.8	3.9	0
Skin Assessment 2	2.7	1.1	1.6	3.8	0
Skin Assessment 3	2.0	0.9	1.1	2.9	0
Nail 1	3.3	1.3	2.1	4.6	0
Nail 2	2.6	1.6	1.0	4.1	0
Nail 3	2.1	1.4	0.7	3.4	1^a^
Nail 4	2.5	1.5	1.0	4.0	0
Nail 5	3.0	1.5	1.5	4.5	0
Skin 1	3.2	1.4	1.9	4.6	0
Skin 2	3.5	1.2	2.2	4.7	0
Skin 3	4.0	1.1	2.9	5.2	1^a^
Skin 4	2.6	1.4	1.2	4.1	0
Hygiene 1	3.7	1.2	2.5	4.9	0
Hygiene 2	4.0	1.1	2.8	5.1	1^a^
Hygiene 3	3.0	1.1	1.9	4.1	0
Movement and Toe Exercise 1	3.4	1.1	2.4	4.5	0
Movement and Toe Exercise 2	3.4	1.3	2.1	4.7	0
Movement and Toe Exercise 3	3.0	1.2	1.8	4.2	0
Consultation 1	2.4	1.2	1.2	3.6	0
Consultation 2	2.6	1.3	1.3	3.9	0

^a^ceiling effect

Results for Cronbach’s alpha are detailed in Table 9. Accordingly, items on Skin Assessment, Nail, Skin, Hygiene, Movement and Toe Exercise, and Consultation had Cronbach’s alpha values of 0.72, 0.67, 0.65, 0.65, 0.73, and 0.63, respectively.

**Table 9:** Cronbach’s alpha values for subscales on practice

Subscale (practice)	Number of items (***n*** = 20)	Cronbach’s α	Mean	SD	Min-to-max value
Skin Assessment	3	0.72	7.6	2.5	3.0–14.0
Nail	5	0.67	13.5	4.7	5.0–25.0
Skin	4	0.65	13.3	3.6	4.0–20.0
Hygiene	3	0.65	10.7	2.7	3.0–15.0
Movement and Toe Exercise	3	0.73	9.9	2.9	3.0–15.0
Consultation	2	0.63	4.9	2.1	2.0–10.0

### Correlation between knowledge and practice scores

The correlation between knowledge and practice scores is presented in Table 10. Accordingly, a significant correlation between overall knowledge and practice scores was observed among both nurses (0.331; *p* = 0.017) and care workers (0.339; *p* = 0.000). Spearman’s rank correlation test showed that the association between knowledge scores and Movement and Toe Exercises was above 0.4 for nurses, while association between knowledge scores and Skin, Hygiene, and Movement and Toe Exercise were above 0.3 for care workers.

**Table 10:** Correlation between knowledge and practice scores among nurses and care workers

*N* = 194						
Subscale (practice)	Nurses (***n*** = 52)			Care workers (***n*** = 142)		
	n	coefficient	***p*** value	N	coefficient	***p*** value
Skin Assessment	52	0.227	0.105	142	0.101	0.236
Nail	52	0.259	0.064	142	0.188	0.026*
Skin	52	0.186	0.186	142	0.313	0.000***
Hygiene	52	0.191	0.176	142	0.350	0.000***
Movement and Toe Exercise	52	0.417	0.002**	142	0.304	0.000***
Consultation	52	0.190	0.178	142	0.235	0.005**
Total	52	0.331	0.017*	140	0.339	0.000***

Spearman’s rank correlation

*p < 0.05

**p < 0.01

*** *p* < 0.001

## Discussion

The results of the present study showed a significant association between foot care knowledge and practices among both nurses and care workers. The purpose of this study was to explore the strengths and weaknesses of both professions with regard to the provision of foot care and to suggest future strategies that improve the level of care within this area. One of features of the present study was the inclusion of care workers among the study participants. Care workers working in in-home service providers have countless opportunities to assess and come in contact with the client. Thus, statements like “he/she is not as usual” by non-nurses should be taken seriously and require follow-up [15].

Our results showed that both nurses and care workers were interested in learning about foot care and observing clients’ foot problems despite having low confidence, insufficient time, and limited foot care education. Indeed, 57 (92%) nurses and 165 (97%) care workers had cared for a mean of 7.9 and 9.5 clients with foot problems a month before the survey.

In contrast to our hypothesis, a significant correlation between working experience and practice scores had been observed, with full-time participants having higher mean scores. Moreover, working experience was significantly associated with practice scores among care workers. This is consistent with results presented in previous studies [19] and could perhaps be attributed to increased chances for foot care practice with greater working hours.

Higher accuracy differences in the early detection of foot problems had been observed between both groups in contrast to our hypothesis. Despite nurses having received more in-depth anatomy and physiology education compared to care workers, only slight differences had been expected given the lack of foot care education in both professions. Previous studies indicated nursing assistant’ detection of early signs of symptom contribute to health care [15, 17]. Older people with low risk of foot problems might be undiagnosed and overlooked; therefore, they need medical help [24] and with high risk may develop worse conditions [25, 26].

Early detection and reporting of foot problems by care workers may lead to early treatment, which could potentially be life-saving. Therefore, enhancing knowledge on early detection among care workers should be emphasized.

The present study found that knowledge on shoes and socks had been lacking among both professions given the lower accuracy rates of related answers. Despite having more opportunities to observe the client’s foot when assisting with the wearing of shoes and socks or bathing, care workers were less aware of foot arches compared to nurses. Inappropriate shoes can cause calluses or corns, as well as toe and arch deformity [27, 28]. This is significant considering that the arch of the foot plays a vital role in balancing or walking. Although the effect of inappropriate footwear on the structure of foot has been extensively studied in other countries [28–33], limited research on the same has been available in Japan.

Nurse and care workers were aware of the protective effects of moisturizers on the skin barrier; however, the 25.5% knowledge difference between both groups regarding skin tears should be emphasized in future foot education programs. Considering the decreased elasticity, dryness, and fragility of older people’ skin, identifying factors that trigger skin tears on their arms and feet can prevent further skin problems. Accordingly, Serra et al. had reported risk factors for skin tears among frail populations [34]. Notably, small stones or objects inside the shoes may lead to skin breakage on the foot. Observing for signs on skin from improper footwear is imperative [35]. Assessment of the skin between the toes and on the heel has also been poor among Japanese studies, unlike those in other countries [36–38]. Skin maceration between the toes may increase the risk for developing cellulitis from fungal infections. Indeed, a hospital based-study in Japan reported fungal infections among older people individuals [39], with another study on older people living at home and in nursing homes also showing the same [40]. Hence, assessing the skin between the toes should be included in a health care provider’s daily routine.

The present study found that more nurses than care workers practiced nail care. However, nurses had the lowest scores for ingrown nail care among the items on nail care practice. Admittedly, foot nail care, particularly nail cutting, among older people individuals can be challenging for both of nurses and care workers. Changes in nail characteristics may be normal and related to the natural aging process. However, nail disorders, including thickened, elongated, and ingrown nails, can be painful and disabling [41]. The MHLW has provided interpretation reports on “Article 17 of the Doctors Act, Article 17 of the Dentists Act and Article 31 of the Public Health Nurses, Midwife Nurse, Nurse Act and related laws and regulations” and listed items, regarding foot care, that are not considered as medical practice as a general rule [42].. Such information would be beneficial for the safe and regulated practice of foot care among nurses and care workers. Learning to use a grinder and toenail clipper requires time and knowledge. Care providers may also learn to use a nail file for reducing nail thickness to some degree or shape the nail edge. Nail disorder not only caused cosmetic problems but also showed negative effects on health-related quality of life and psychological problems [13, 43]. Thus, nail care among older people individuals greatly contributes toward maintaining and improving quality of life.

Nurses and care workers had close mean scores for Movement and Toe Exercise. The present study included prevention of sedentary behavior and toe exercises in the general definition of foot care. A wide range of studies outside Japan have shown that sitting for long durations without standing every hour may cause adverse effects on the body [44–46]. Hence, monitoring sedentary time and promoting hourly standing among older people individuals should theoretically be promoted. However, this becomes challenging for nurses and care workers due to time constraints and the need for careful observation relate to safety.

The current study identified several challenges for future programs. Firstly, time constraints continue to be a universal issue for the nursing profession. Evidence has clearly shown that workload and access to equipment are among the challenges nurses and care workers face, which could lead to insufficient time allotted toward caring for clients [47, 48], most of whom are vulnerable. When caring for several clients at one time, nurses and care workers observe them carefully and assist with walking or bathing, taking extreme caution due to the risk for falls. To account for this situation, efficient and comprehensive “hands-on” foot care tools, which can be learned and implemented quickly during regular working hours, can be developed for nurses and care workers. Previous studies can also be used as reference [36, 48, 49].

Secondly, the lack of foot care education in the school curriculum as well as in the work field has hindered foot care practice in Japan. According to our study, 78.7% (48) of nurses and 75.7% of care workers (128) stated that foot care manuals are necessary. Moreover, the results presented herein showed that foot care knowledge came from various sources, with some care providers not even knowing the source (Table 3). Hence, a certain structured system for foot care education should be incorporated into the current academic curriculum. Detecting foot problems or providing foot care for particular foot problems among older people individuals has remained challenging. Stolt et al. stressed the necessity of having regular, organized continuing education for all professional nurses engaged in clinical practice [11].

Gaining knowledge and practical experience through education or training sessions has been shown to foster confidence. Lack of confidence may affect the delivery of care [50]. Self-efficacy and confidence has often been associated with self-care behavior among patients with diabetes [51]. Both the nurse’s and care worker’s confidence may supplement foot self-care insufficiency among older people due to aging. Coping with nail thickness or reducing edema however may require further foot care education and training.

Thirdly, the lack of foot care specialists has hindered appropriate treatment of foot problems in Japan. Accordingly, Japan does not provide a national license for foot care specialists or foot care doctors equivalent to a podiatrist or pomologist. Moreover, the present study suggested that the current consultation system is lacking due to an absence of podiatry referral system in Japan compared to other countries [52, 53]. While the incorporation of referral recommendations may be influenced by many factors [54], communication channels represent the strength of an organization. Considering that nurses and care workers in in-home service providers do not immediately receive orders from doctors, unlike those in the hospital, they may have more autonomy over decisions regarding further referral after observing or assessing the foot problem.

The present study has explored the requirements for future foot care programs targeting nurses and care workers. Given the various current limitations, new approaches toward enhancing knowledge and practice among nurses and care workers need to be developed and exercised in the future. Additional large-scale studies on nurses and care workers in in-home service providers will be essential. However, researchers need to formulate strategies that address potential participation bias with the current Japanese working situation.

### Study limitations

The participants included herein were collected using cluster sampling. Thus, once a field manager of a service center expressed willingness to respond to this study, nurses and care workers were more likely to cooperate with the study. Nonetheless, we need to accumulate evidence and provide reasons for the achieved response rates. Although 530 participants were initially targeted, this number could not be met due to time and budget constraints. Nonetheless, the final sample size was determined to be statistically appropriate. Noncertified care workers had several different types of certificates, the differences in which could not be analyzed due to the small number of participants. Moreover, some nurses worked as home care providers, which allowed them more time to assess the skin and toes of their clients. The time allocated for foot care might differ depending on provider types and their roles. To properly account for and analyze potential differences, future studies need to include a large enough sample from each type of provider. The present study obtained Cronbach’s alpha values of 0.63–0.73 for all subscales on practice, which could have been attributed to the number of items included in each subscale.

## Conclusions

It was clarified that the nursing care staff’s interest and willingness to learn about foot care and the actual situation of foot care education are inversely proportional to the actual situation of foot care education. In addition, the findings provide suggestion that specific foot care education is needed for items that had a statistical difference between both occupations, including early detection for foot problems and skin tears, and questions with a low accuracy rate or low average value even if there was no statistical difference between the groups.

## Data Availability

All data generated or analyzed during this study are included in this published article and its supplementary information files. The datasets supporting the conclusion of this article are available from the corresponding authors.

## Appendix

### Japanese insurance system

Japan has two types of insurances for older individuals in terms of access to care, medical insurance, and long-term care insurance.

#### Medical insurance

In Japan, all people are supposed to have health insurance, which is known as universal health insurance. People use medical insurance to prepare for medical treatment or examination due to illness or injury. All people regardless of age enroll into public medical insurance. The cardholder is responsible for 10–30% of the treatment cost (typically 30%); however, the rate of burden varies depending on age and income.

Medical insurance is categorized into three types. When the burden of medical cost becomes high due to the long duration of hospitalization or treatment, the limit of payment burden is set known as “high cost medical expense benefit” [55–57].

**Table:**

Type of medical insurance	For the people to use
National health insurance	Self-employed or for the unemployed or others.
Employee’s health insurance for employed people	Health insurance from their employer such as health insurance association, mutual aid association, or association/union administrated health insurance
Medical care system for the elderly aged ≥75 years	Aged ≥75 years

NOTE: People who have trouble making a living are excluded from national health insurance. The medical cost of these individuals is covered as medical assistance in the frame of public assistant system

#### Long-term care insurance

The second insurance type is the long-term care insurance (LTCI) that is a mandatory public program run by the municipal government and specified district in Tokyo. It is paid extra by all Japanese citizen aged ≥40 years. LTCI can be claimed starting at the age of 65 or over years (category 1) or at the age of 40–64 years for those with specific diseases such as amyotrophic lateral sclerosis, cerebrovascular diseases, e.g. (category 2), and applicants for care services must be screened. Those who are eligible to use the LTCI system are categorized into one of the seven care need levels: two support levels (1 and 2) and care level 1 through 5 [58]. The cardholder will be responsible for 10–30% of the nursing service cost. There is a payment limit depending on the category. Therefore, the nursing care services are selected to not exceed the limit.

According to current calculations based on statistics from the MHLW as of March 2019, approximately 18% individuals aged ≥65 years are people certified for the LTCI [59]. Following reasons are considered regarding why approximately 82% of the population aged ≥65 years is not eligible for the services of LTCI. The actual number of individuals in need of home-based care remains unknown.

**Table:**

They do not to apply the LTCI.	
They may have been denied to receive the system due to a nonqualifying condition or they may have been categorized into the precare group.	
Those aged ≥65 years or whose family may not have sufficient knowledge or information about the LTCI system.	
Those aged ≥65 years or whose family is only willing to receive hospital care.	
Those aged ≥65 years or whose family is not willing to accept care besides the family members or acquaintances.	

## Abbreviations

MHLW: Ministry of Health, Labor and Welfare
NHI: National Health Insurance
LTCI: Long-Term Care Insurance
CVI: Content Validity Index
I-CVI: Item Content Validity Index
RN: Registered nurse
LPN: Licensed practical nurse
